# Supplementary material for: Species-specific ribosomal RNA-FISH identifies interspecies cellular-material exchange, active-cell population dynamics and cellular localization of translation machinery in clostridial cultures and co-cultures
Source: mSystems. 2024 Sep 10;9(10):e00572-24. doi: 10.1128/msystems.00572-24 (PMC11495018; doi:10.1128/msystems.00572-24)
Supplement: Supplemental Figures — Fig. S1 to S19. [file msystems.00572-24-s0001.docx]

**Supplementary material for:**

**Species-specific ribosomal RNA-FISH identifies interspecies cellular-material exchange, active-cell population dynamics and cellular localization of translation machinery in clostridial cultures and co-cultures**

John D. Hill^a^ & Eleftherios T. Papoutsakis^a,#^

^a^ Department of Chemical and Biomolecular Engineering & the Delaware Biotechnology Institute, University of Delaware, 590 Avenue 1743, Newark, DE 19713, USA

Author Contact Information:

John Hill (jdhill@udel.edu)

Eleftherios T. Papoutsakis ([epaps@udel.edu](mailto:epaps@udel.edu))

John D. Hill http://orcid.org/0000-0001-6127-3238

Eleftherios Terry Papoutsakis http://orcid.org/0000-0002-1077-1277

#Corresponding Author Information

Eleftherios T. Papoutsakis

590 Avenue 1743, Newark, DE 19713, USA

(302) 831-8376

[epaps@udel.edu](mailto:epaps@udel.edu)


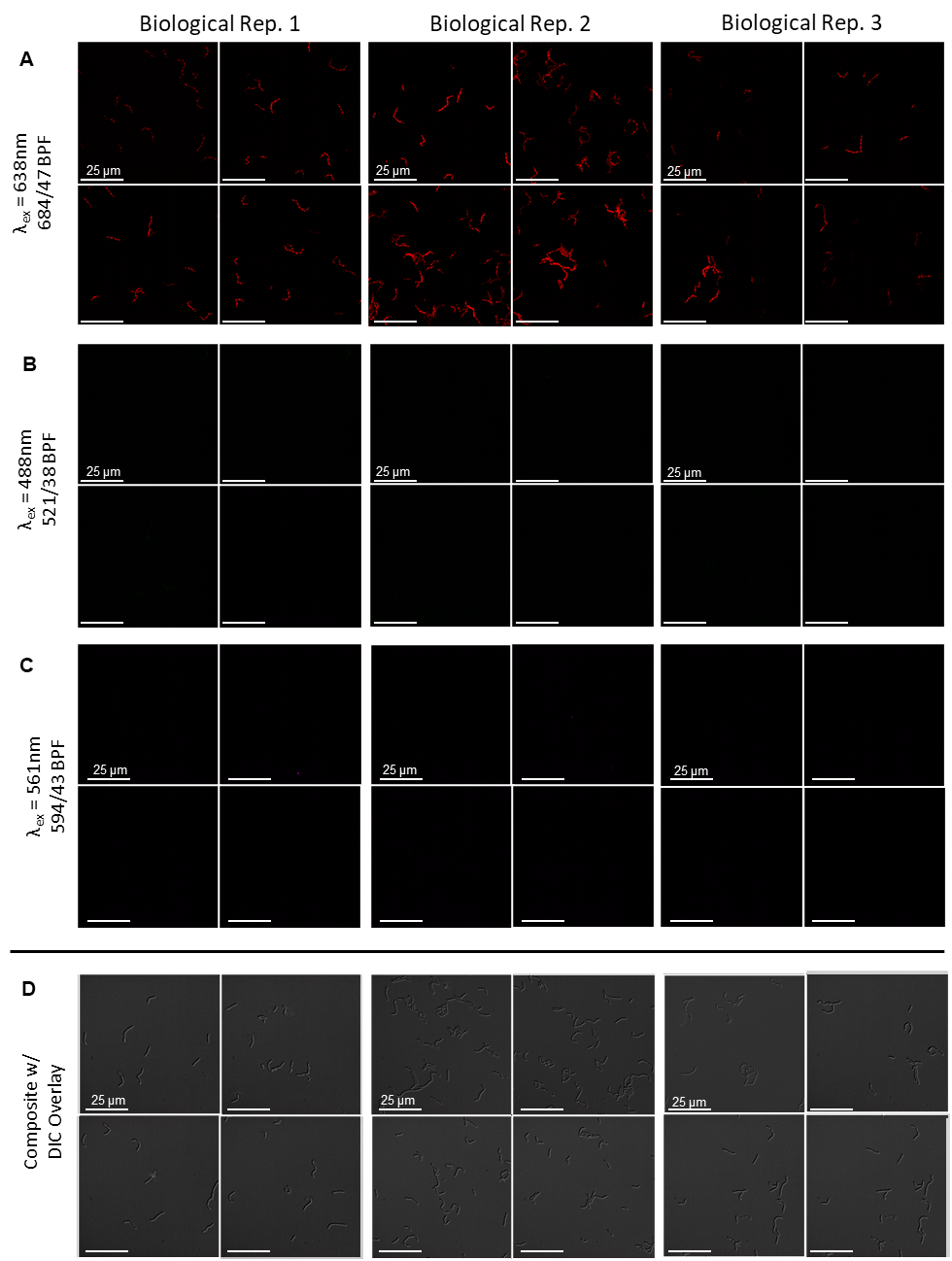


**Fig S1** Fluorescent microscopy images of *C. ljungdahlii* from FIG 1.E. **(A-C)** 4 random fields from each of 3 labelled (incubated with all three sets of probes simultaneously) biological replicates were imaged via the far-red channel (λ_ex_=638 nm, BPF = 684/47, specific to ClosLjun) **(A),** the green channel (λ_ex_=488 nm, BPF = 521/38, specific to ClosKluy) **(B),** and the yellow channel (pseudo-colored magenta) (λ_ex_=561 nm, BPF = 594/43, specific to ClosAcet**) (C).** Only red fluorescence, coming from ClosLjun, could be detected. (**D)** Composite images (all three channels stacked) with DIC overlay reveal the absence of autofluorescence in the unlabeled cells. High resolution TIFF images can be found on *figshare* in the collection - <https://figshare.com/account/home#/collections/7381186>


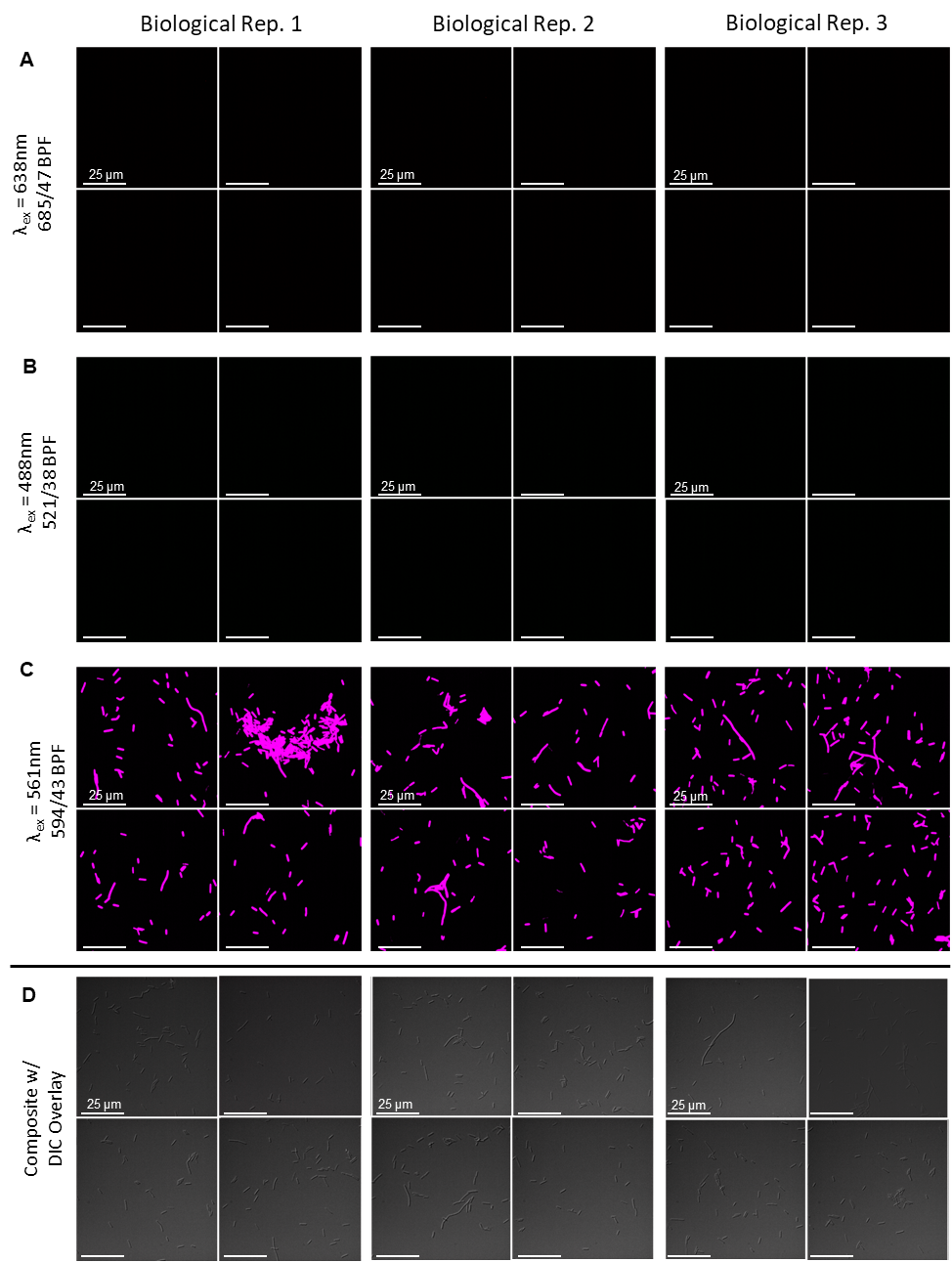


**Fig S2** Fluorescent microscopy images of *C. acetobutylicum* from FIG 1.D. **(A-C)** 4 random fields from each of 3 labelled (incubated with all three sets of probes simultaneously) biological replicates were imaged via the far-red channel (λ_ex_=638 nm, BPF = 684/47, specific to ClosLjun) **(A),** the green channel (λ_ex_=488 nm, BPF = 521/38, specific to ClosKluy) **(B),** and the yellow channel (pseudo-colored magenta) (λ_ex_=561 nm, BPF = 594/43, specific to ClosAcet) **(C).** Only magenta fluorescence, coming from ClosAcet, could be detected. **(D)** Composite images (all three channels stacked) with DIC overlay reveal the absence of autofluorescence in the unlabeled cells. High resolution TIFF images can be found on *figshare* in the collection - <https://figshare.com/account/home#/collections/7381186>


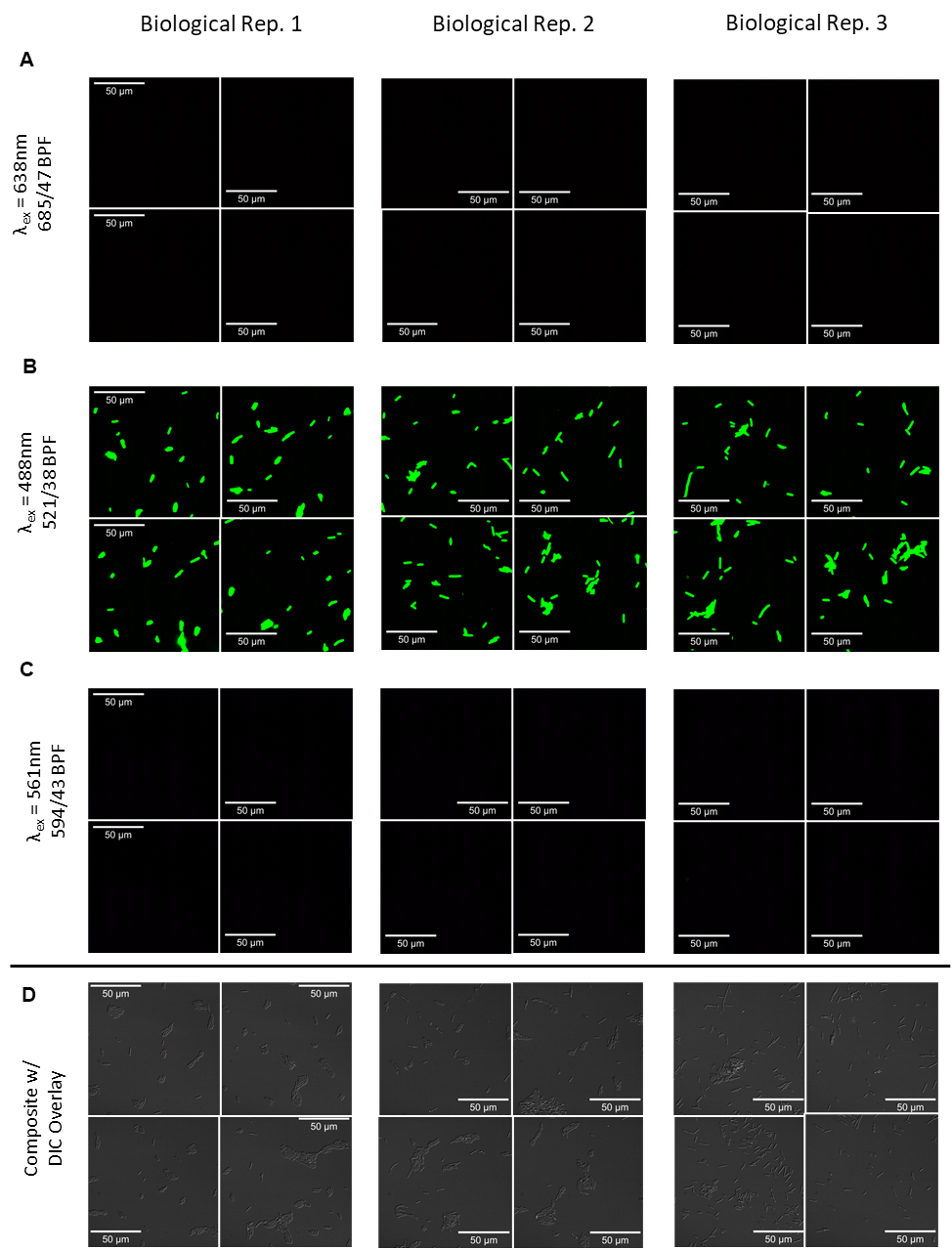


**Fig S3** Fluorescent microscopy images of *C. kluyveri* from FIG 1.F. **(A-C)** 4 random fields from each of 3 labelled (incubated with all three sets of probes simultaneously) biological replicates were imaged via the far-red channel (λ_ex_=638 nm, BPF = 684/47, specific to ClosLjun) **(A),** the green channel (λ_ex_=488 nm, BPF = 521/38, specific to ClosKluy) (B), and the yellow channel (pseudo-colored magenta) (λ_ex_=561 nm, BPF = 594/43, specific to ClosAcet) **(C).** Only green fluorescence, coming from ClosKluy, could be detected. **(D)** Composite images (all three channels stacked) with DIC overlay reveal the absence of autofluorescence in the unlabeled cells. High resolution TIFF images can be found on *figshare* in the collection - <https://figshare.com/account/home#/collections/7381186>


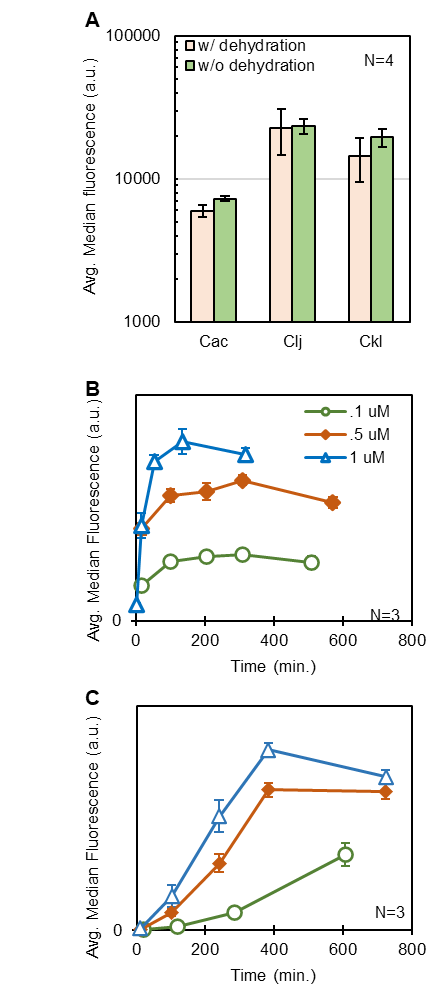


**FIG S4** *In-solution* rRNA-FISH method optimization. **(A)** The average median fluorescence of 4 biological replicate populations of *C. acetobutylicum* (Cac), *C. ljungdahlii* (Clj), and *C. kluyveri* (Ckl) were measured with or without an additional prehybridization dehydration step. No statistically significant change was observed. Error bars represent a single standard deviation above and below the mean in all figures. **(B and C)** The probe concentration and hybridization duration were optimized. In **(B)**, 3 biological replicates of exponentially growing *C. acetobutylicum* were fixed and incubated with three different concentrations of ClosAcet. The average median fluorescence of the three replicates (in arbitrary units, a.u., linear y axis, indexed at 0) was tracked over 10 hours. **(C)** Demonstrates the analogous experiment performed in *C. ljungdahlii* with ClosLjun.


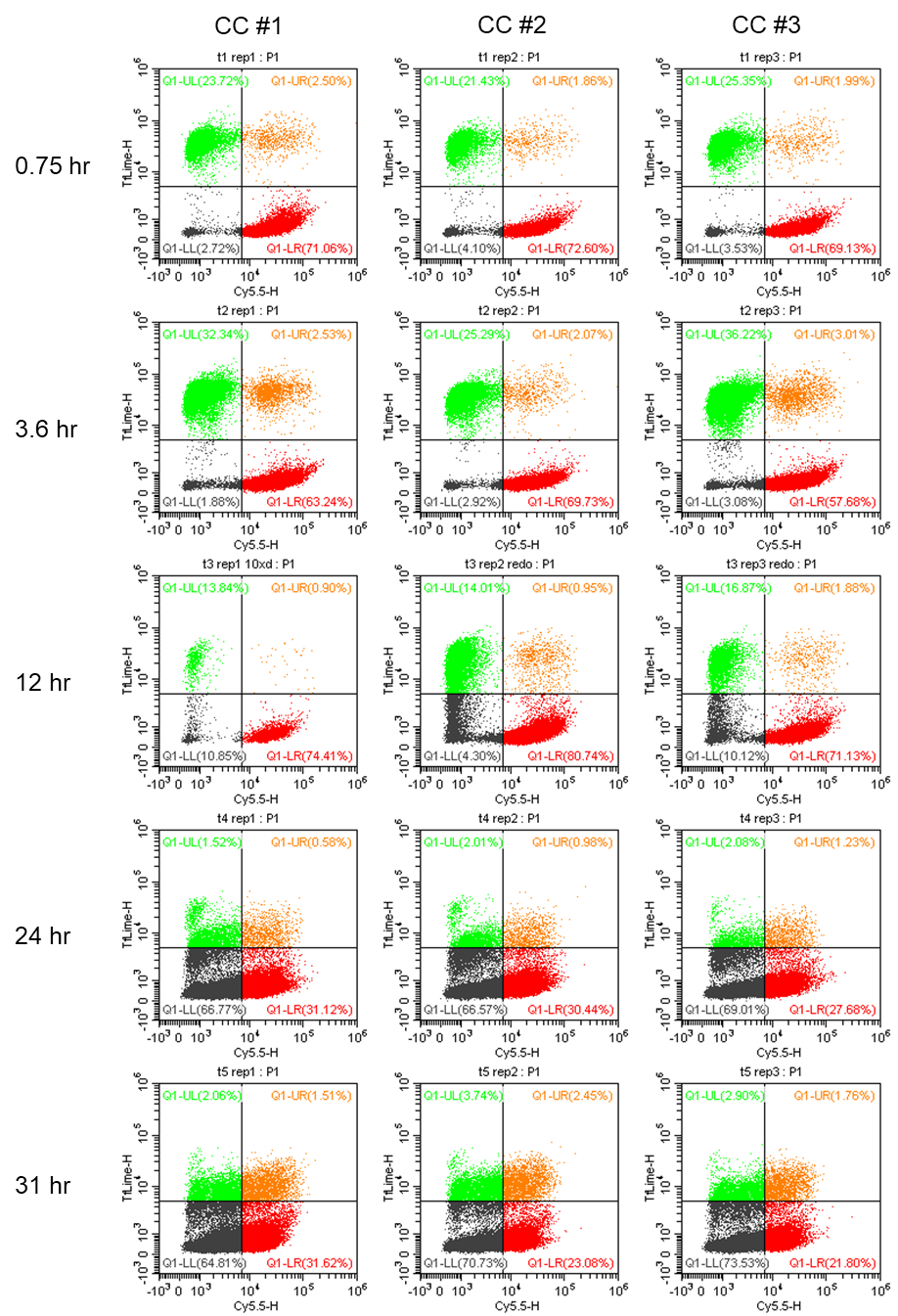


**Fig S5** Flow cytometry dot-plots used to determine subpopulations and hybrid cell frequency in the co-culture between *C. kluyveri* and *C. ljungdahlii*.


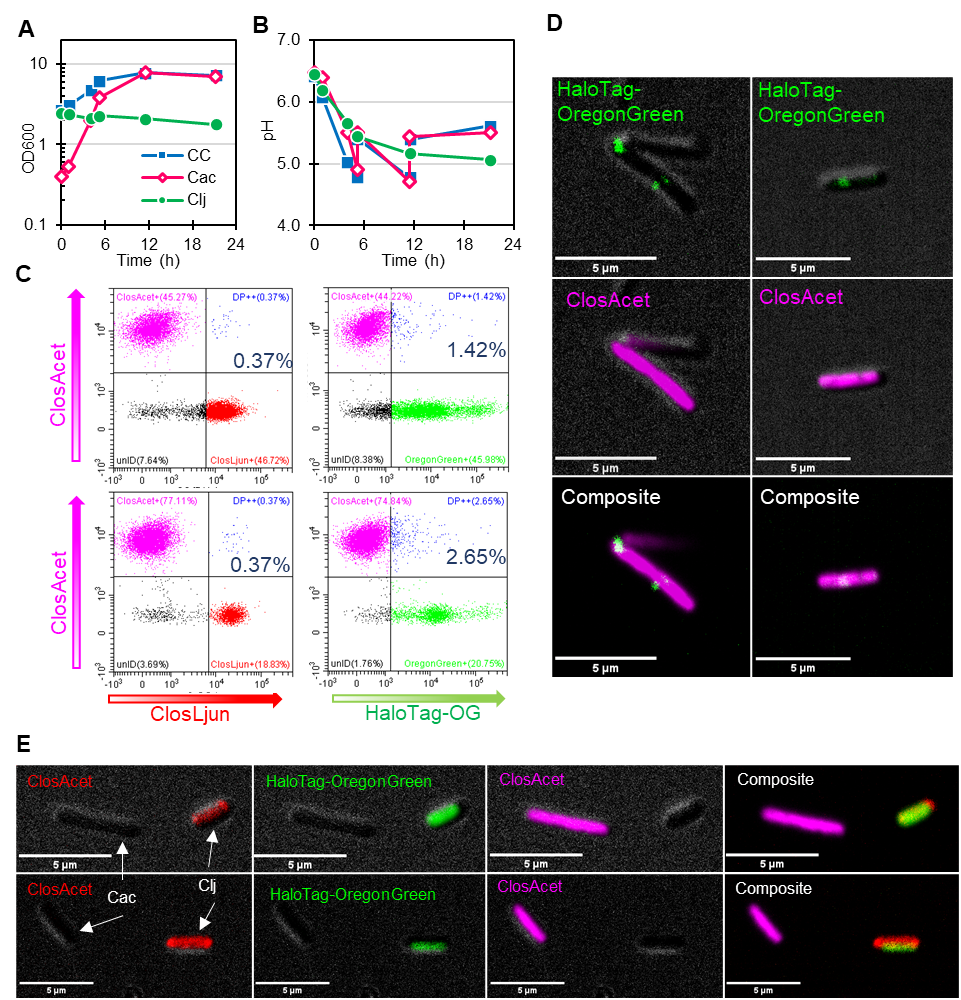


**Fig S6** *C. ljungdahlii-*p100ptaHalo (Clj) to *C. acetobutylicum* (Cac) in co-culture. At 1, 4 and 11 hours, the culture was sampled and labelled with ClosAcet, ClosLjun, and HaloTag-OregonGreen (HaloTag-OG). **(A)** The OD_600_ and **(B)** pH of the co-culture and mono-culture controls. **(C)** Flow-cytometry dot plots measured the frequency of double positives with signals from ClosAcet and either HaloTag-OregonGreen (right column) or *C. ljungdahlii’s* ClosLjun labelled rRNA (left column). **(D)** Fluorescent microscopy images of hybrid cells from the 4-hour timepoint containing rRNA from *C. acetobutylicum* (magenta) and HaloTag-OregonGreen (green) from *C. ljungdahlii*-p100ptaHALO. HaloTag-OregonGreen fluorescence forms puncta. For comparison, **(E)** shows confocal microscopy images of the non-hybrid phenotype from the same time point. Dim DIC images are provided in the background to show cell location. *C. acetobutylicum* exhibits strong ClosAcet (magenta) fluorescence. *C. ljungdahlii-*p100ptaHalo exhibits both green and red signal from the HaloTag-OregonGreen complex and ClosLjun, respectively. **Explanation of results:** Charubin et al. had hypothesized that different macromolecules could be exchanged between *C. acetobutylicum* and *C. ljungdahlii* at different rates based on flow cytometric data that showed substantially faster (total) RNA exchange than protein exchange (1). Since the rRNA-FISH probes are compatible with fluorescent proteins, we were able to revisit this hypothesis by tracking the rate of protein and rRNA exchange simultaneously in a co-culture of *C. acetobutylicum* and *C. ljungdahlii*. A co-culture was performed between *C. ljungdahlii*-p100ptaHALO and *C. acetobutylicum* ATCC with an R-value of roughly 1.7, where the R-value is the starting ratio of *C. ljungdahlii* to *C. acetobutylicum* based on OD_600_ and preculture volume (2). Though *C. ljungdahlii-*p100ptaHALO precultures contained erythromycin, a translation inhibitor which targets the large ribosomal subunit, the cells were thoroughly washed prior to inoculation into the co-culture to prevent inhibition of *C. acetobutylicum* growth. Mono-culture controls were performed simultaneously (Fig. S6A). After the initial drop in pH caused by the accumulation of acids (Fig. S6B), the pH was maintained by addition of NaOH to prevent acid death (1). During the first 11 hours, samples were labelled with the HaloTag ligand OregonGreen, ClosAcet and ClosLjun, then analyzed via flow cytometry and microscopy (Fig. S6C, S6D, and S6E). OregonGreen was chosen as the fluorescent HaloTag ligand since its fluorescent signal does not overlap with ClosAcet (Cy3, yellow emitting, pseudo-colored magenta for clarity) and ClosLjun (Cy5.5, far-red emitting). *C. ljungdahlii* has two fluorescent labels, the HaloTag-OregonGreen complex and the ClosLjun probe (Fig. S6E). *C. acetobutylicum’s* cytoplasm is labelled only by the ClosAcet probe, (Fig. S6E). Population gating for HaloTag-OregonGreen signal was based on the mono-culture controls (Fig. S7). Population gating for ClosAcet and ClosLjun were based on the control experiments in Fig 1D and 1E and verified against mono-culture controls (Fig. S7). Either the coexistence of the ClosAcet and HaloTag-OregonGreen signals or the overlapping of the ClosAcet and ClosLjun signals would constitute a hybrid event. The largest fraction of hybrid events was found at 4 hours (Fig. S6C, Fig. S7). At this time point, 2.65% of events exhibited ClosAcet/HaloTag-OregonGreen double fluorescence, while only 0.37% of events exhibited ClosAcet/ClosLjun double fluorescence. Other authors have pointed out that random cell aggregation may skew flow-cytometric results (3). Our data, however, demonstrate that hybrid events attributed to cell fusion are not artifacts of cell aggregation during culturing or labelling. If the ‘hybrid events’ were predominantly due to cell aggregates containing both species, we would expect those events to emit signals from ClosAcet, ClosLjun, and HaloTag-OregonGreen since the vast majority of cells attributed to the *C. ljungdahlii* population emit signals from both ClosLjun and HaloTag-OregonGreen (Fig. S8). The majority of hybrid events emit signal only from ClosAcet and HaloTag-OregonGreen and are, therefore, not aggregates. Moreover, aggregates are likely to form during repeated centrifugation and incomplete resuspension, but our simplified protocol obviates that risk. It is worth noting that microscopy images of hybrid cells, HaloTag fluorescence arose primarily from puncta representing probably several dozens to several hundreds of spatially associated HaloTag proteins (Fig. S6D). We recognize that these puncta may arise simply from reagent precipitates, but we did not observe any such precipitates in the microscopy for Fig. S1, S2, and S3. We hypothesize that these events primarily represent *C. acetobutylicum* cells which have acquired functional HaloTag proteins rather than *C. ljungdahlii* cells which have acquired *C. acetobutylicum* ribosomes based on the strong ClosAcet signal coming from these cells. Our findings are different from those of Charubin et al.’s findings that supported the hypothesis that RNA exchange occurs faster than protein exchange (1). This is likely due to differences in measurement techniques. Charubin et al.’s method labels, using a dye, total RNA and not only rRNA. Fluorescence from rRNA-FISH represents assembled and largely actively translating ribosomes. Free mRNA and tRNA are much smaller molecules than ribosomes, and thus, compared to ribosomes, would be exchanged faster through cellular fusion events (1) due to enhanced molecular mobility.

1. Charubin K, Modla S, Caplan JL, Papoutsakis ET. 2020. Interspecies Microbial Fusion and Large-Scale Exchange of Cytoplasmic Proteins and RNA in a Syntrophic Clostridium Coculture. mBio 11:10.1128.

2. Charubin K, Papoutsakis ET. 2019. Direct cell-to-cell exchange of matter in a synthetic Clostridium syntrophy enables CO2 fixation, superior metabolite yields, and an expanded metabolic space. Metabolic Engineering 52:9-19.

3. Bäumler M, Schneider M, Ehrenreich A, Liebl W, Weuster‐Botz D. 2021. Synthetic co‐culture of autotrophic Clostridium carboxidivorans and chain elongating Clostridium kluyveri monitored by flow cytometry. Microbial Biotechnology 15:1471-1485.


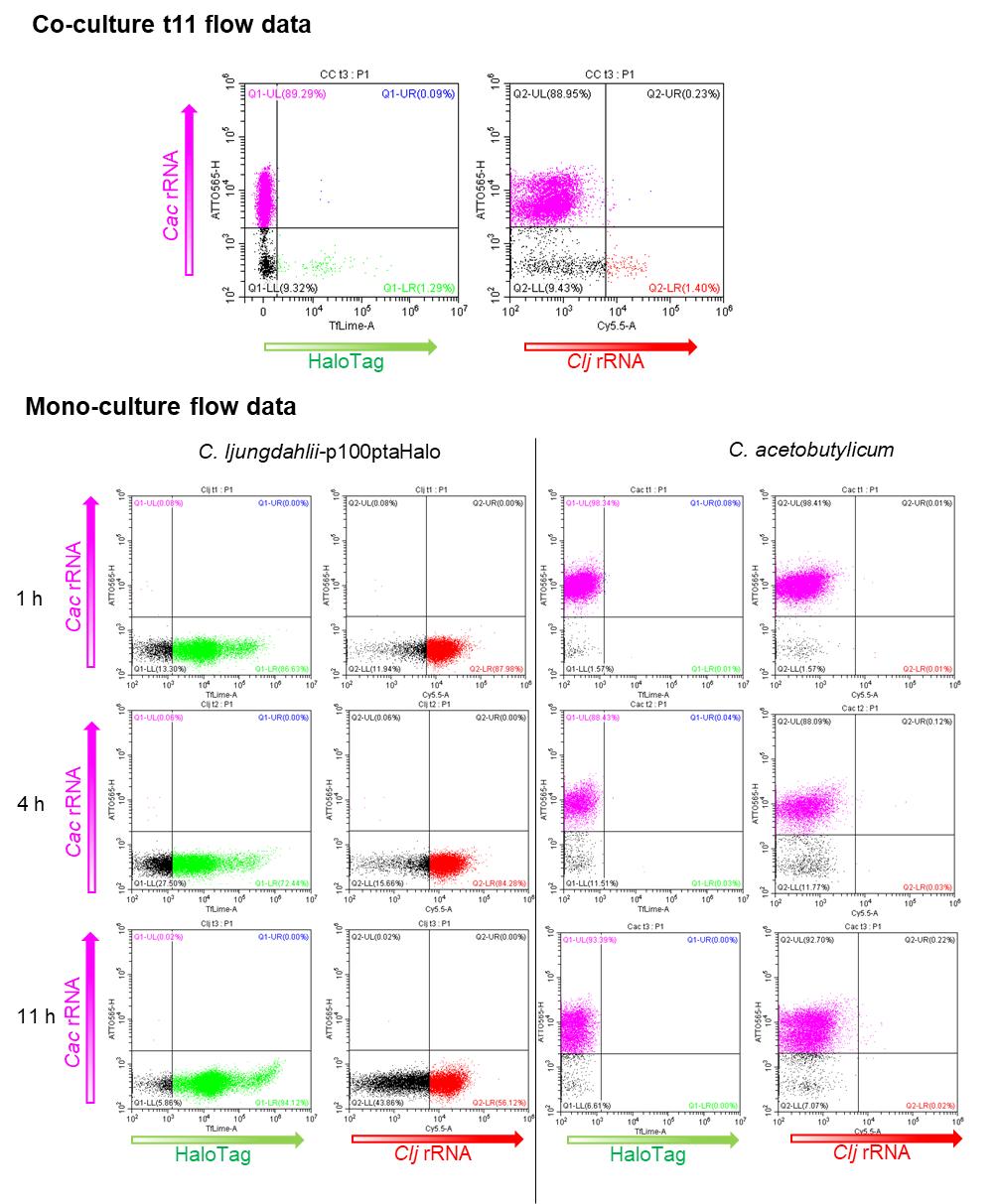


**Fig S7** The 11-hour time point flow cytometry dot-plots and the pure-culture controls for the co-culture in Fig. S6. Double positive cells are essentially non-existent for either species across all timepoints.


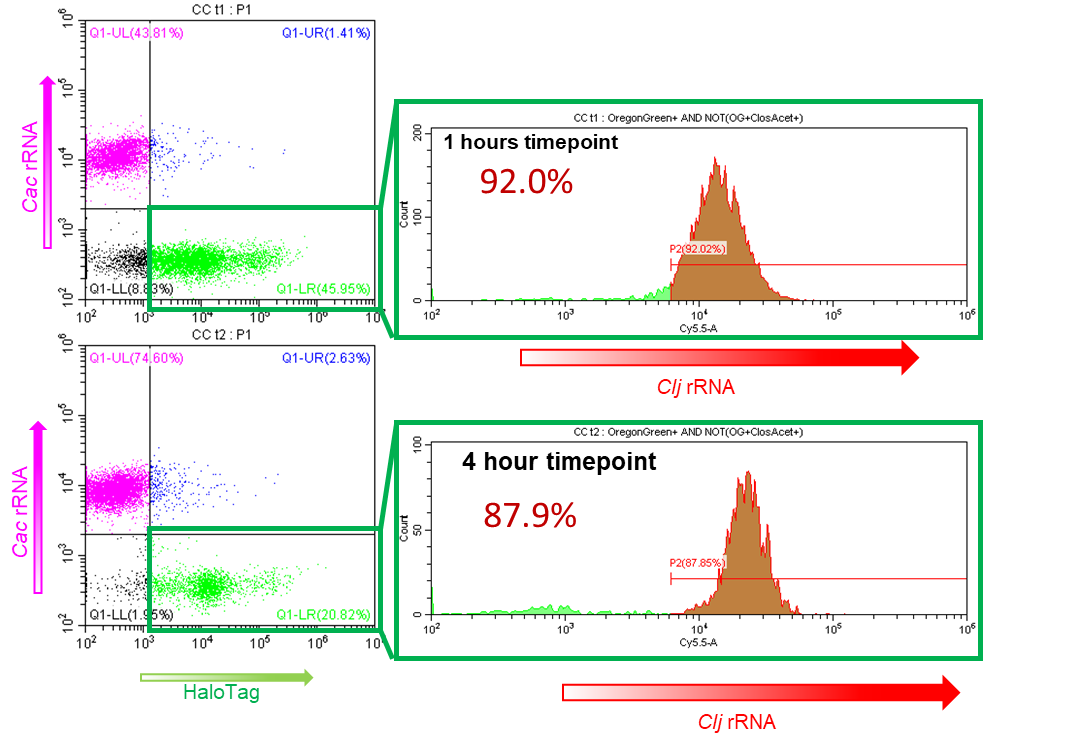


**Fig S8** Prevalence of red fluorescent cells (i.e. high fluorescence on the ClosLjun channel) among green-fluorescent cells (excluding the hybrid subpopulation). This demonstrates that nearly all green labelled cells are double positive for the ClosLjun probe.


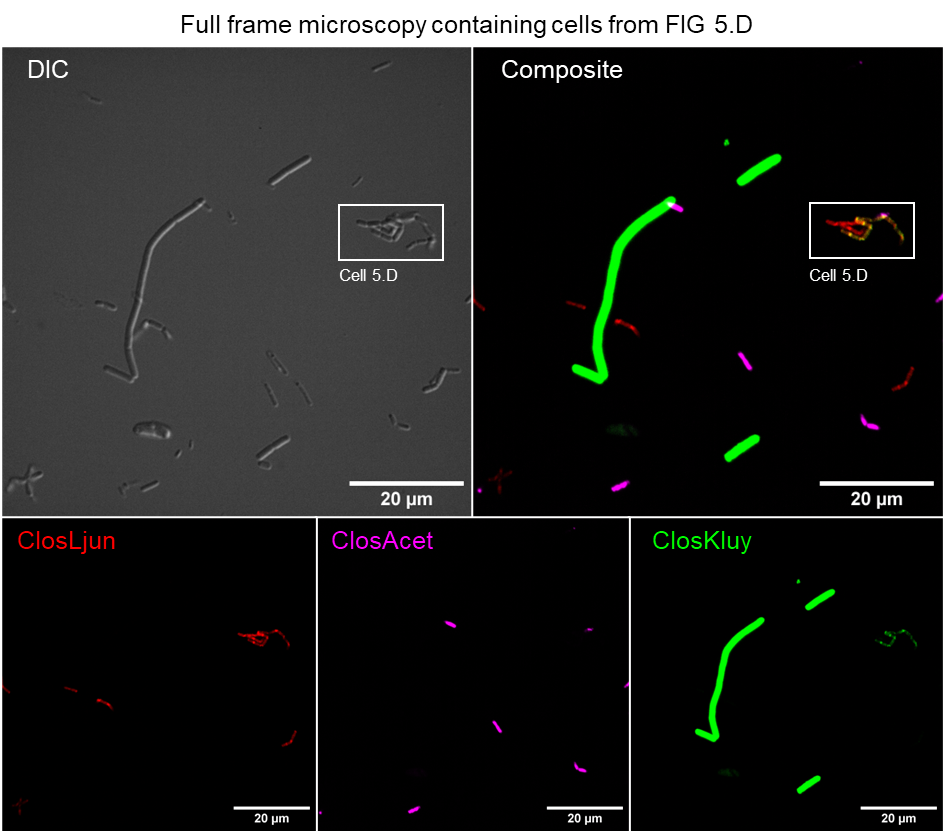


**Fig S9** Full Frame images of the cells depicted in Fig. 5D. In the Top row, the DIC and the composite fluorescent image are presented, with the individual fluorescent channels comprising the bottom row.


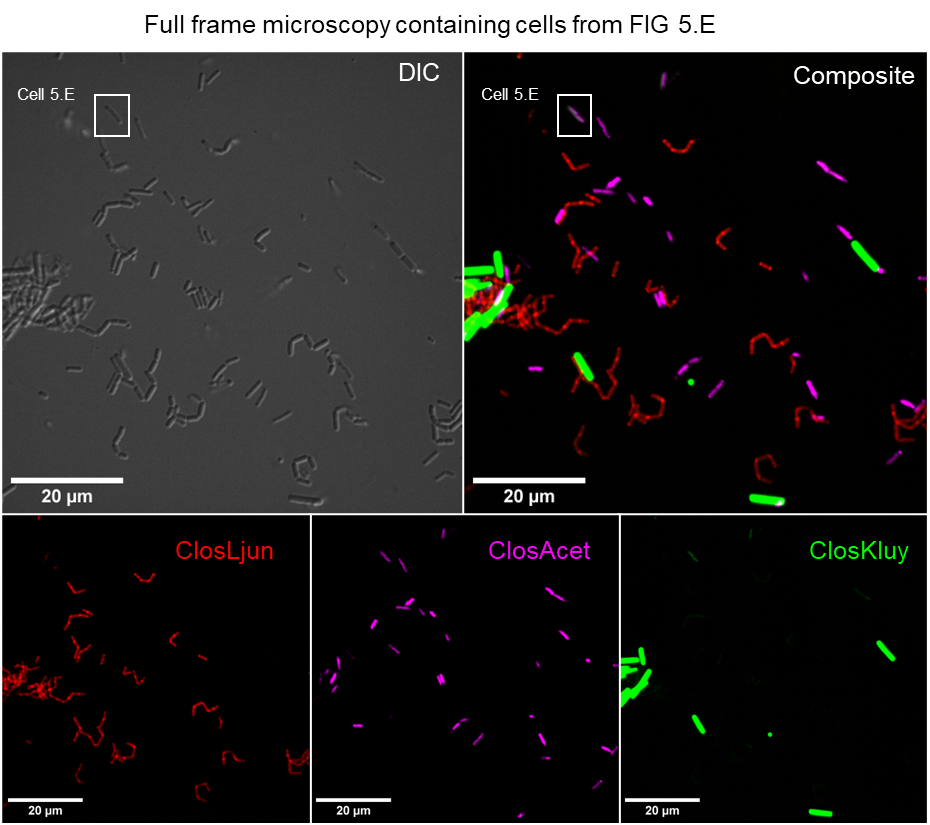


**Fig S10** Full Frame images of the cells depicted in Fig. 5E. In the Top row, the DIC and the composite fluorescent image are presented, with the individual fluorescent channels comprising the bottom row.


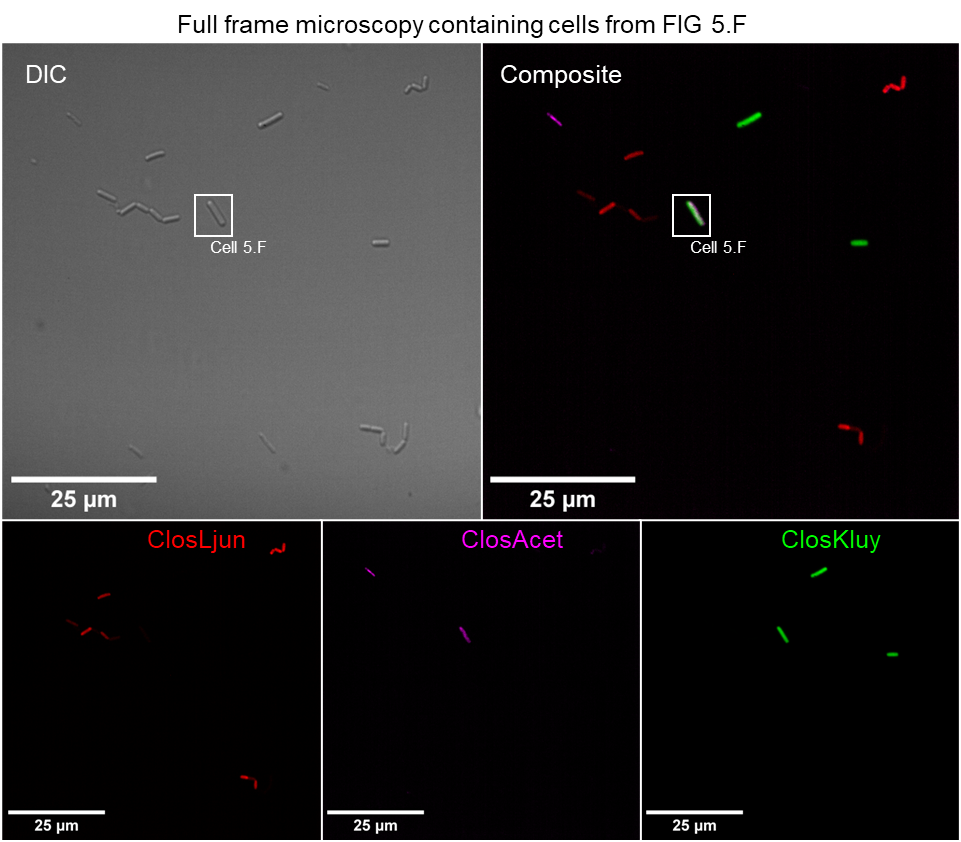


**Fig S11** Full Frame images of the cells depicted in Fig. 5F. In the Top row, the DIC and the composite fluorescent image are presented, with the individual fluorescent channels comprising the bottom row.


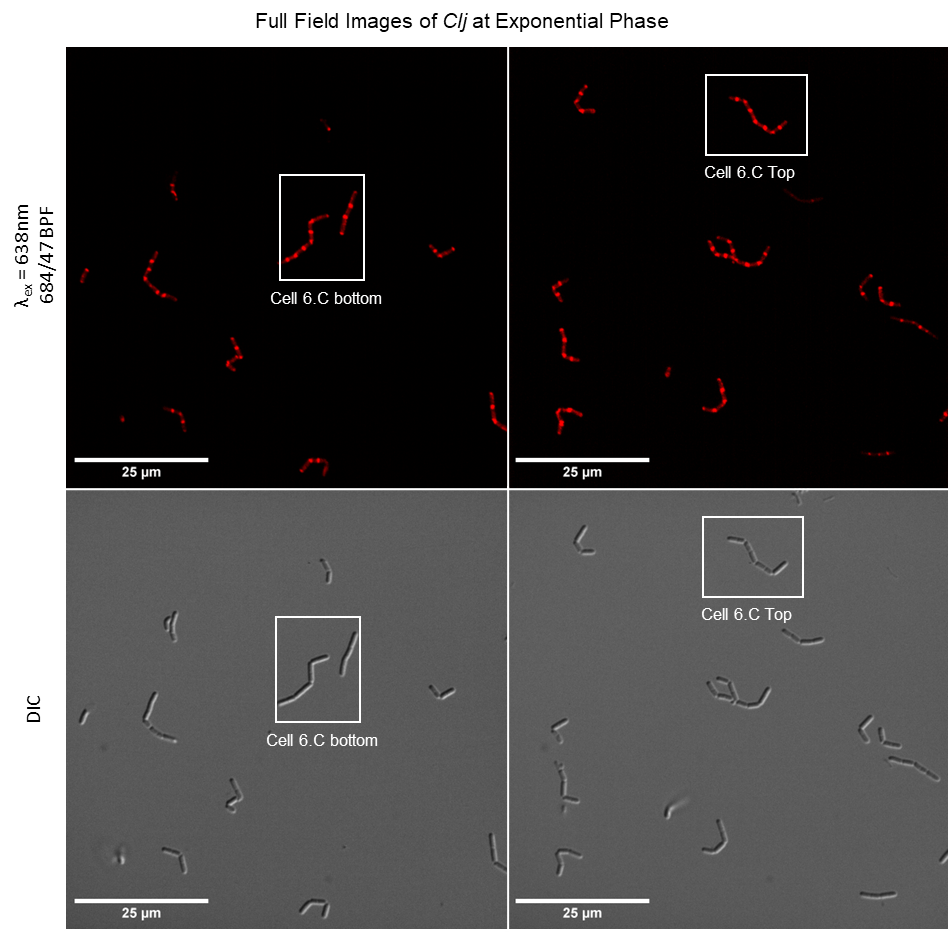


**Fig S12** Full frame microscopy images of the exponential phase cells from Fig. 6C. Fluorescent above and DIC below.


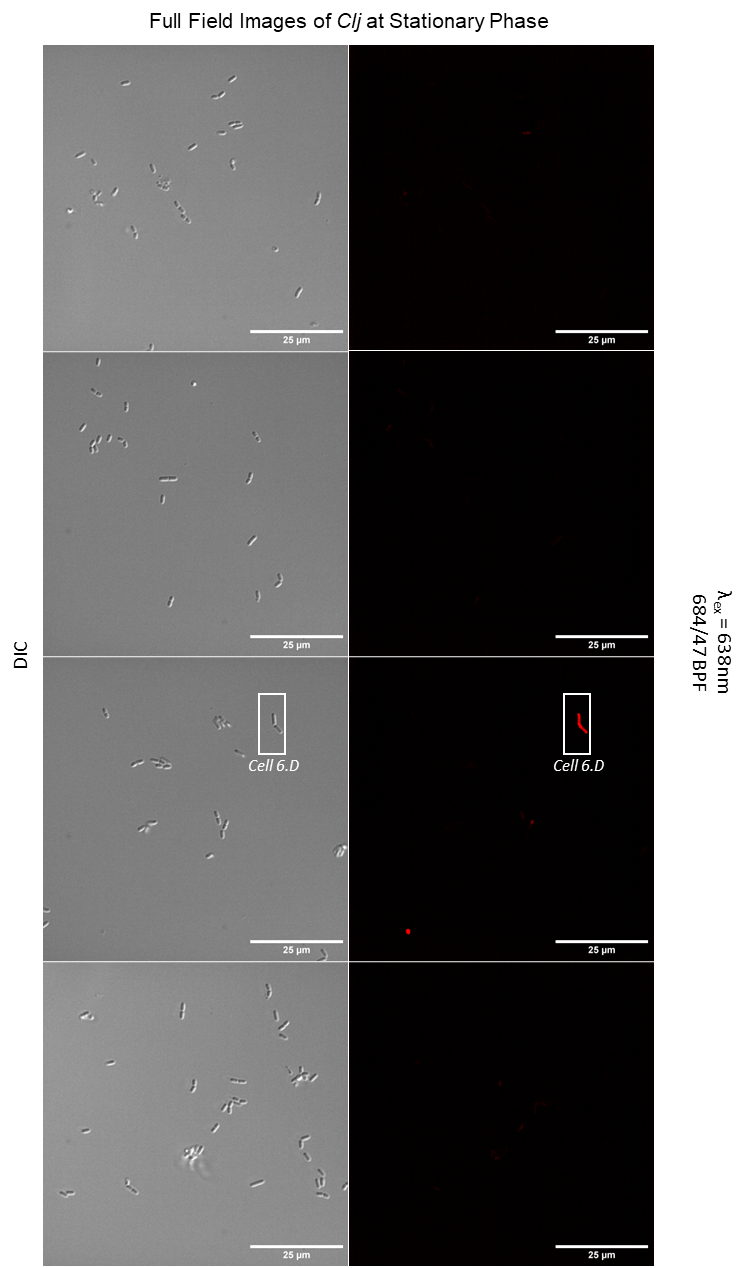


**Fig S13** Full frame microscopy images of the stationary phase cells from Fig. 6D. Fluorescent right and DIC left.


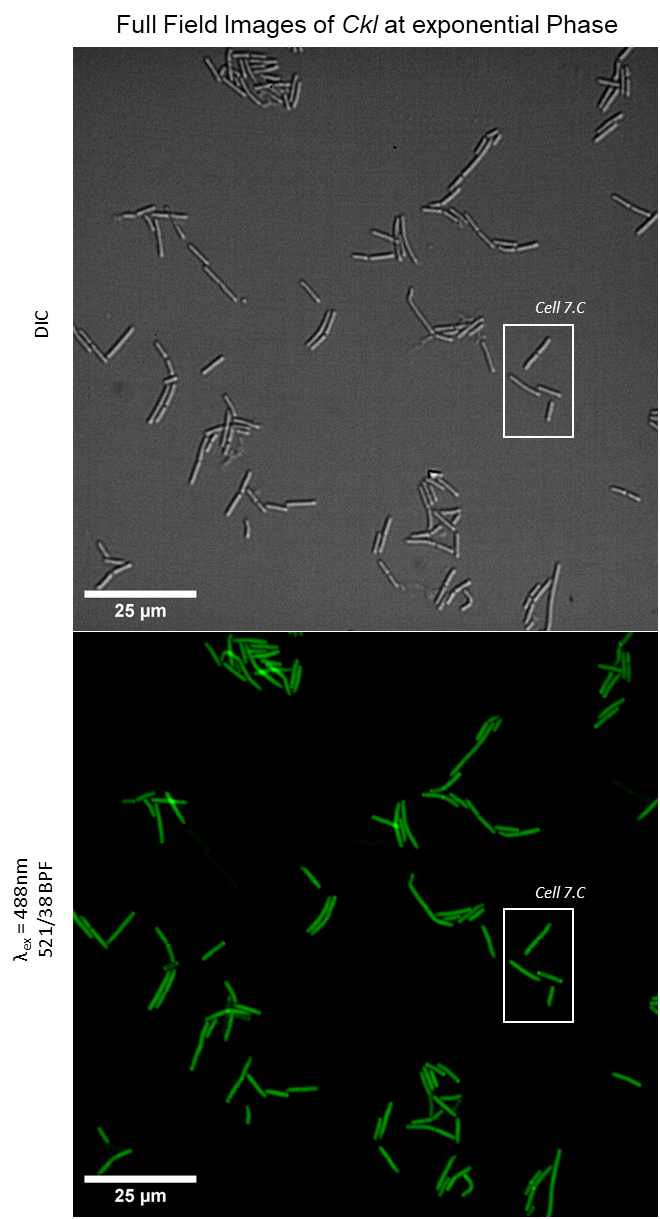


**Figure S14.** Full frame microscopy images of the exponential phase cells from Fig. 7C. Fluorescent below and DIC above.


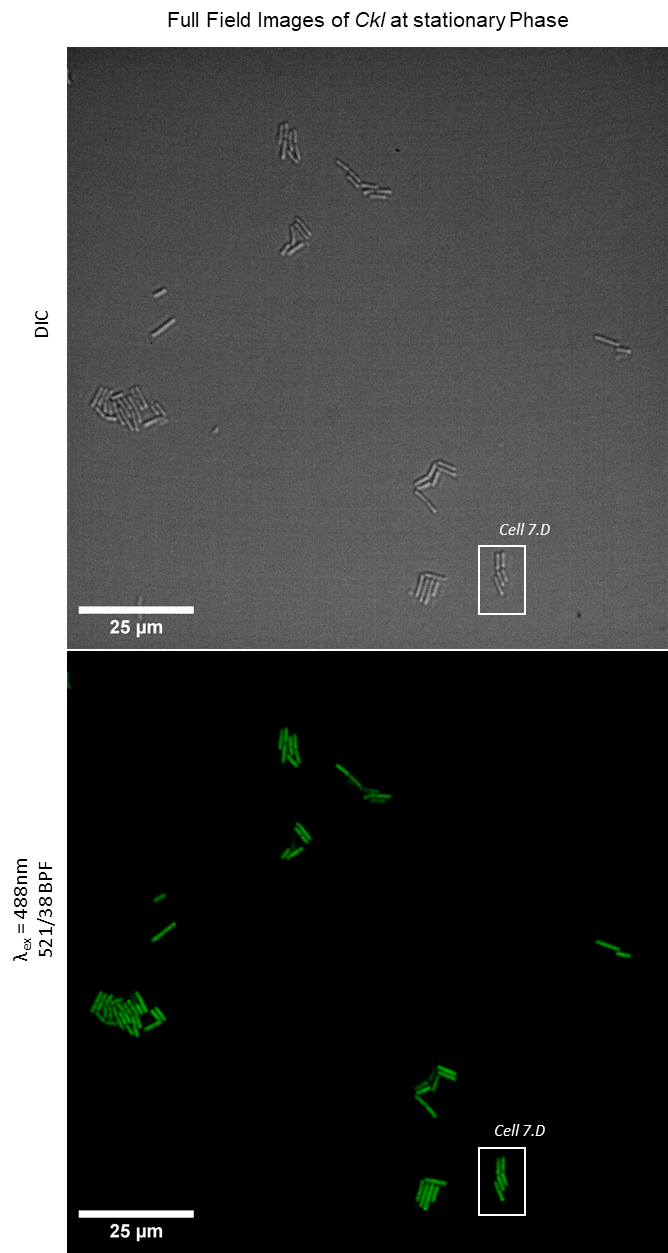


**Figure S15.** Full frame microscopy images of the stationary phase cells from Fig. 7D. Fluorescent below and DIC above.


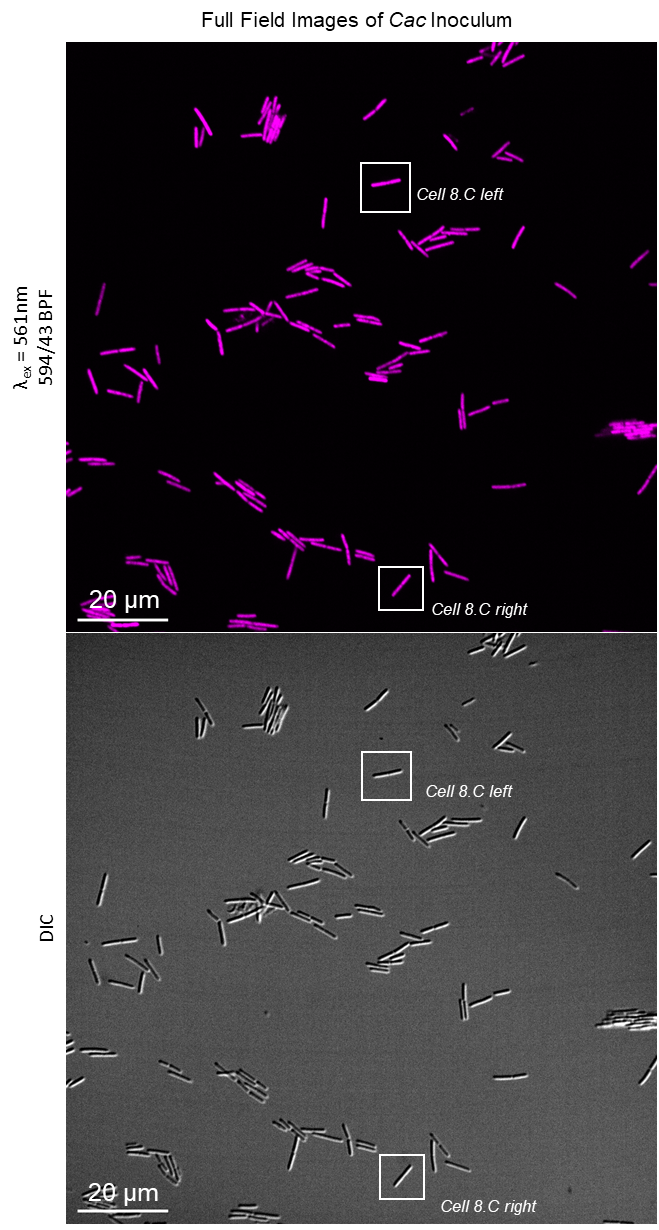


**Fig S16** Full frame microscopy images of the inoculum from Fig. 8C. Fluorescent above and DIC below.


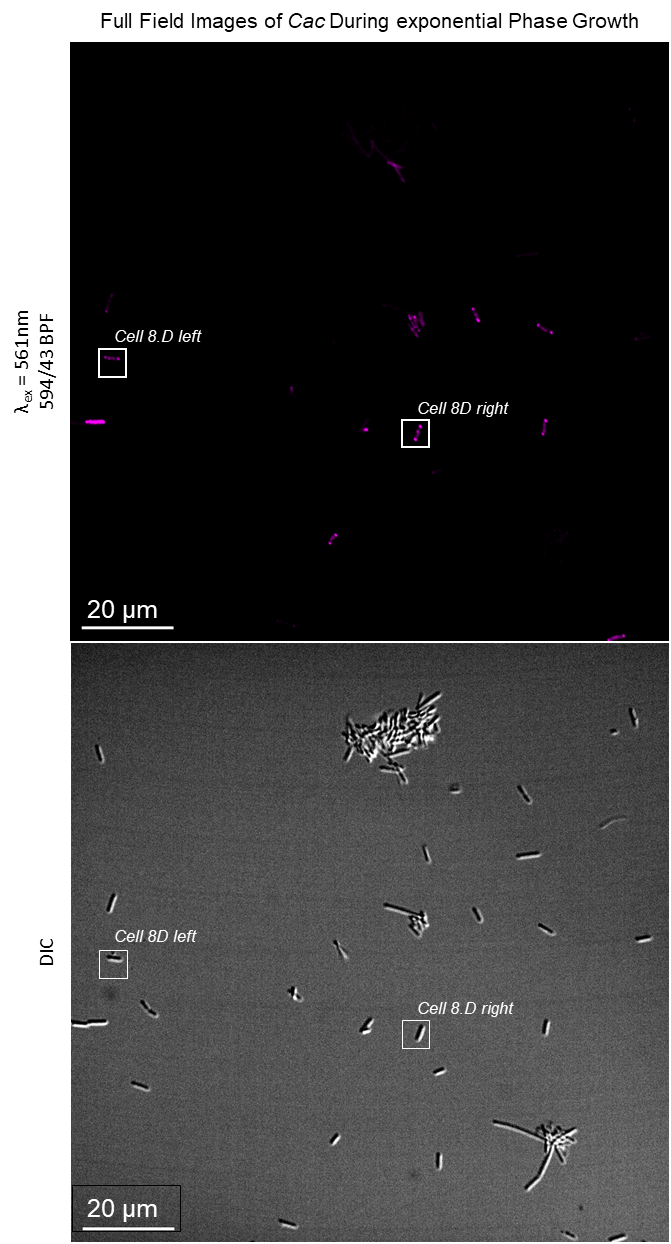


**Fig S17** Full frame microscopy images of the exponential phase cells from Fig. 8D. Fluorescent above and DIC below.


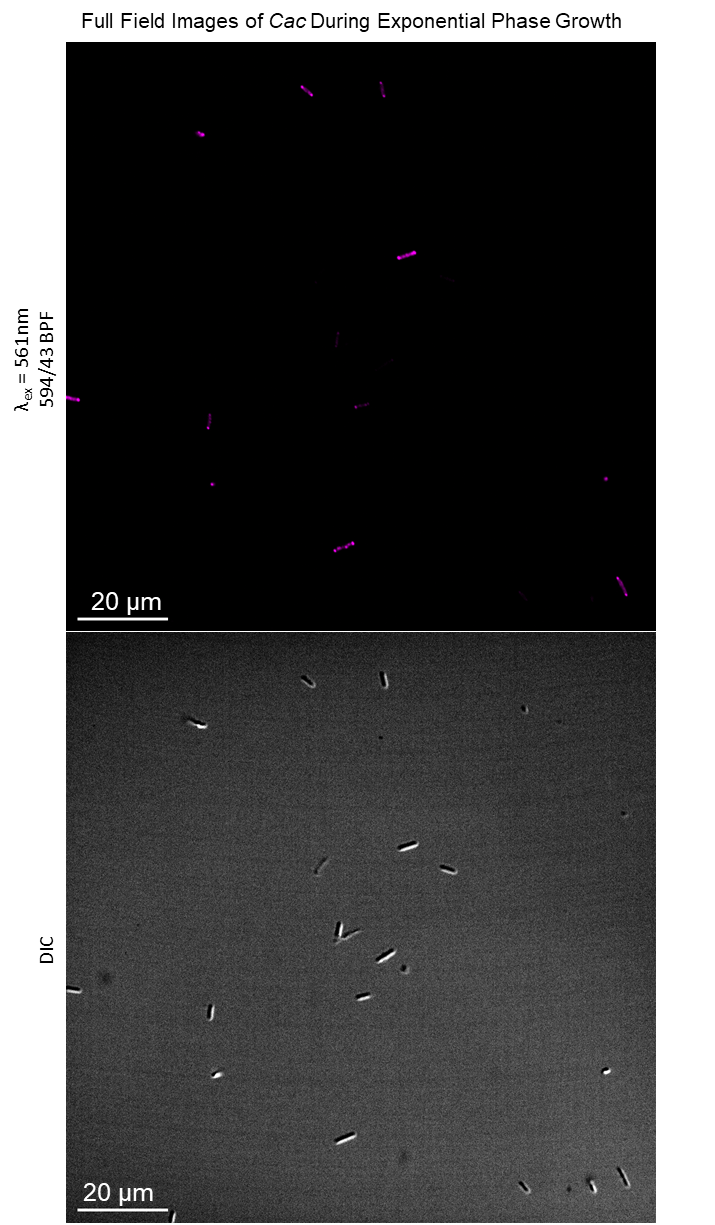


**Fig S18** Additional Full frame microscopy images of the exponential phase cells from Fig. 8D. Fluorescent above and DIC below. This second image (in addition to Fig. S17) is included because the cell density is lower.


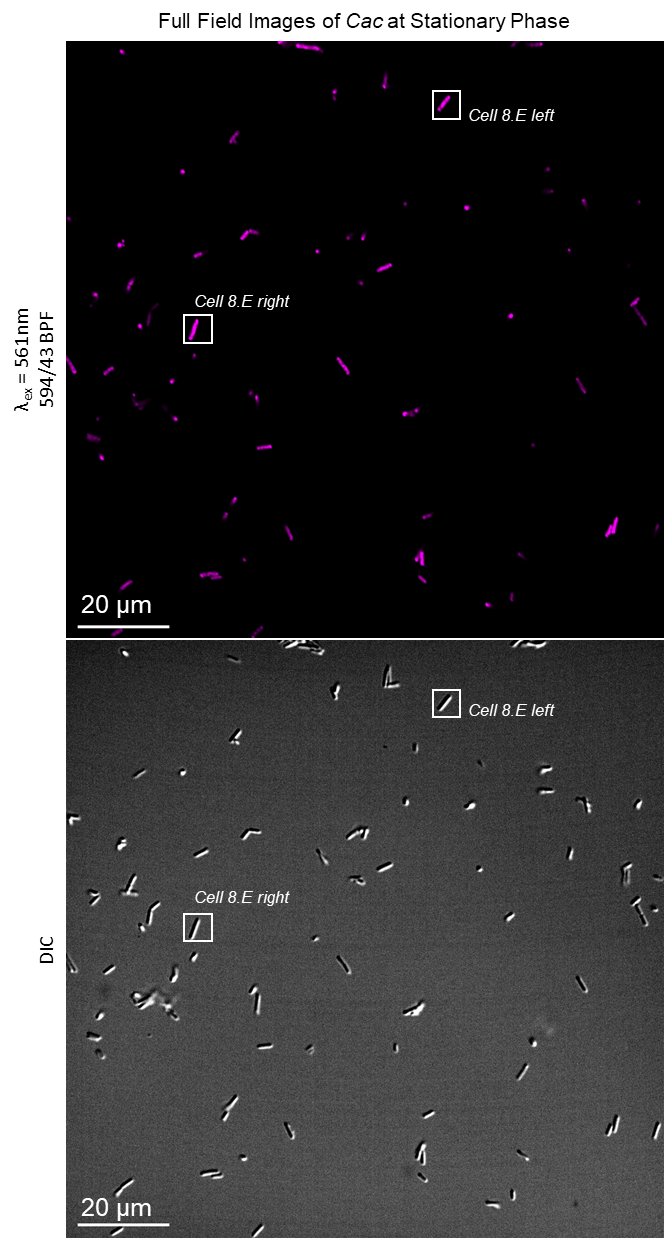


**Fig S19** Full frame microscopy images of the stationary phase cells from Fig. 8E. Fluorescent above and DIC below.
