# Supplementary material for: Species-specific ribosomal RNA-FISH identifies interspecies cellular-material exchange, active-cell population dynamics and cellular localization of translation machinery in clostridial cultures and co-cultures
Source: mSystems. 2024 Sep 10;9(10):e00572-24. doi: 10.1128/msystems.00572-24 (PMC11495018; doi:10.1128/msystems.00572-24)
Supplement: Supplemental Tables — Tables S1 to S4. [file msystems.00572-24-s0002.docx]

**Supplementary tables for:**

**Species-specific ribosomal RNA-FISH identifies interspecies cellular-material exchange, active-cell population dynamics and cellular localization of translation machinery in clostridial cultures and co-cultures**

John D. Hill^a^ & Eleftherios T. Papoutsakis^a,#^

^a^ Department of Chemical and Biomolecular Engineering & the Delaware Biotechnology Institute, University of Delaware, 590 Avenue 1743, Newark, DE 19713, USA

Author Contact Information:

John Hill (jdhill@udel.edu)

Eleftherios T. Papoutsakis ([epaps@udel.edu](mailto:epaps@udel.edu))

John D. Hill http://orcid.org/0000-0001-6127-3238

Eleftherios Terry Papoutsakis http://orcid.org/0000-0002-1077-1277

#Corresponding Author Information

Eleftherios T. Papoutsakis

590 Avenue 1743, Newark, DE 19713, USA

(302) 831-8376

[epaps@udel.edu](mailto:epaps@udel.edu)

**Table S1**

Frequency of species as identified by flow cytometry of samples from monocultures which were labelled with all three species-specific probes simultaneously. This data was used to determine if the probes had sufficient specificity. Data was generated from the histograms in Fig. 1 of the manuscript. The values presented represents the average of 3 biological replicates.

|  | **Fluorescent Probe/Channel** | | |
| --- | --- | --- | --- |
|  | ClosAcet | ClosLjun | ClosKluy |
| ***C. acetobutylicum*** | 90.55% | 0.02% | 0.00% |
| ***C. ljungdahlii*** | 0.26% | 94.49% | 0.06% |
| ***C. kluyveri*** | 0.01% | 0.22% | 98.32% |

**Tables S2**

Frequency of species as identified by flow cytometry of monoculture samples which were not labelled with any probes. This data was used to determine if autofluorescence would contribute to identification of species. Data was generated from the histograms in Fig. 1 of the manuscript. The values presented represents the average of 3 biological replicates.

|  | **Fluorescent Probe/Channel** | | |
| --- | --- | --- | --- |
|  | ClosAcet | ClosLjun | ClosKluy |
| ***C. acetobutylicum*** | 0.04% | 0.00% | 0.00% |
| ***C. ljungdahlii*** | 0.00% | 0.18% | 0.00% |
| ***C. kluyveri*** | 0.01% | 0.00% | 0.05% |

**Table S3**

Probes used in this study

| Probe | Target Organism | Sequence | Fluorophor | Melting Temp | Reference |
| --- | --- | --- | --- | --- | --- |
| ClosAcet | *C. acetobutylicum* | 5’-CCTGACGGAACTGCTTCC-3’ | Cy3 | 68.6 | This study |
| ClosLjun | *C. ljungdahlii* | 5’-CGCCACTACTTCCTAGTC-3’ | Cy5.5 | 71.9 | This Study |
| ClosKluy | *C. kluyveri* | 5’-GCGGACTCCCCTTCAAAG-3’ | AlexaFluor 488 | 69.4 | Schneider et al. |

**Table S4**

NaCl and EDTA concentration in washing buffer

| **Formamide % in Hyb. Buff.** | **NaCl conc.** | **EDTA conc.** |
| --- | --- | --- |
| 10 | 0.45 M | omitted |
| 20 | 0.215 M | 5 μM |
| 30 | 0.102 M | 5 μM |
| 40 | 0.046 M | 5 μM |
| 50 | 0.018 M | 5 μM |
